# Mice engineered to mimic a common Val66Met polymorphism in the BDNF gene show greater sensitivity to reversal in environmental contingencies

**DOI:** 10.1016/j.dcn.2018.05.009

**Published:** 2018-05-30

**Authors:** Angela Vandenberg, Wan Chen Lin, Lung-Hao Tai, Dorit Ron, Linda Wilbrecht

**Affiliations:** a Neuroscience Graduate Program, University of California, San Francisco, CA, 94158, USA; b Department of Psychology, University of California, Berkeley, CA, 94720, USA; c Department of Neurology, University of California, San Francisco, CA, 94158, USA; d Helen Wills Neuroscience Institute, University of California, Berkeley, CA, 94720 USA

**Keywords:** Learning, Flexibility, Updating, Neurotrophin, Executive function

## Abstract

• A common human polymorphism in the gene that encodes brain derived neurotrophic factor (BDNF), Val66Met, is considered a marker of vulnerability for mental health issues and has been associated with cognitive impairment. An alternate framework has been proposed in which “risk alleles” are reinterpreted as “plasticity alleles” that confer vulnerability in adverse environments and positive effects in neutral or positive environments (Belsky et al., 2009). These frameworks produce divergent predictions for tests of learning and cognitive flexibility. Here, we examined multiple aspects of learning and cognitive flexibility in a relatively new BDNF Val66Met mouse model (BDNF Val68Met, Warnault et al., 2016), including multiple choice discrimination and reversal, go/no-go learning and reversal, and appetitive extinction learning. We found that mice homozygous for the Met allele show more efficient reversal learning in two different paradigms, but learn at rates comparable to Val homozygotes on the multiple choice discrimination task, a go/no-go task, and in appetitive extinction. Our results dissociate reversal performance from go/no-go learning and appetitive extinction and support the plasticity allele framework that suggests BDNF Met carriers are potentially more sensitive to changes in the environment.

## Highlights

• A new line of mice,Val68Met, mimic human BDNF Val66Met polymorphism.
• New knock-in BDNF Met mice reverse more efficiently than Val in two separate tasks.
• Supports theory that BDNF Met allele confers greater sensitivity to the environment.
• Reversal performance can be dissociated from go/no-go and extinction performance.
• Phenotypes differ between newer and older BDNF Val66Met mouse models.

## 1 Introduction

A common variant in the gene that encodes brain derived neurotrophic factor (BDNF) (Egan et al., 2003) is of considerable interest due to the extensive role of BDNF in neurodevelopment and plasticity. This common single nucleotide polymorphism encodes a valine (Val) to methionine (Met) substitution at codon 66 at the prodomain of the gene (Val66Met) in humans which is analogous to codon 68 in mice. The Met substitution at this codon confers a trafficking deficit which results in decreased activity dependent release of BDNF (Chen et al., 2006). This polymorphism occurs in 20–30% of the human population and has been linked to deficits in select forms of memory (Egan et al., 2003), as well as susceptibility to psychiatric disorders (Angelucci et al., 2005; Chen et al., 2006; Frielingsdorf et al., 2010; Gratacos et al., 2007; Joffe et al., 2009), including substance abuse (Cheng et al., 2005; Duncan, 2012; Biskupska et al., 2013; Greenwald et al., 2013). Differences in activity dependent release of BDNF may alter the time course or trajectory of neural circuit development by many routes (Bath and Lee, 2006; Pattwell et al., 2012; Vandenberg et al., 2015; Wang et al., 2015; Jing et al., 2017).

Multiple studies of human subjects have indicated that BDNF Met carriers have compromised cognitive function (Egan et al., 2003; Tsai et al., 2004; Bath and Lee, 2006; Miyajima et al., 2008; Schofield et al., 2009). However, there have also been reports of benefits to executive functions (Erickson et al., 2008; Ventriglia et al., 2002; Matsushita et al., 2005; Beste et al., 2010a, b; Gajewski et al., 2011, 2012; Alfimova et al., 2012; Getzmann et al., 2013). In one example, Beste et al. found that carriers of the Met allele had fewer false alarms on a go/no-go task, which correlated with larger no-go-N2 event related potentials (ERPs) in Met carriers (Beste et al., 2010a). A recent meta-analysis focused on cognitive ability, memory, executive function, visual processing skills and cognitive fluency find mixed effects for each domain and concludes there is no evidence for association between the polymorphism and a cognitive phenotype (Mandelman and Grigorenko, 2012).

It is possible that task type, age, and individual environment may contribute to differences across studies generating mixed effects in meta-analyses. There is a growing body of literature suggesting that the BDNF 66Met allele may confer greater sensitivity to the environment, garnering benefits when it is positive and more negative outcomes when it is adverse (Belsky et al., 2009; Drury et al., 2012; Gerritsen et al., 2012). This is consistent with a proposal to reframe risk alleles as plasticity alleles (Belsky et al., 2009). Here, we examined learning and cognitive flexibility in a battery of tests in a mouse model of the human BDNF Val66Met polymorphism to test its effect on executive function when age and environment are relatively controlled. We chose to examine go/no-go learning due to reports of differences in humans (Beste et al., 2010a). We examined extinction learning due to differences observed in a study of another BDNF Val66Met mouse line (Briand et al., 2012). We examined multiple-choice discrimination learning as way to test learning under enhanced cognitive load. Finally, we examined reversal, in go/no-go and in multiple choice learning, to test cognitive flexibility in face of a changing environment. Using the risk allele framework one would predict that learning and cognitive flexibility would be impaired in BDNF 66Met mice, potentially due to cognitive impairment or inefficiency. Using a plasticity allele framework, one would predict that learning would be intact and flexibility might even be enhanced due to greater sensitivity to the environment (not because of any optimal or adaptive value for flexibility in any given context).

We found that animals homozygous for the Met allele from a recently published BDNF Met knock in line (Warnault et al., 2016) showed no differences in the three different forms of learning, go/no-go, appetitive extinction, and multiple-choice discrimination. However, homozygous Met mice from this line do show significantly more efficient reversal performance in two separate tasks, suggesting greater flexibility in response to changing contingencies. These data are consistent with the ‘plasticity allele’ theory that BDNF 66Met allele may confer greater sensitivity to the environment.

## 2 Materials and methods

### 2.1 Animals

All animal procedures were approved by the Ernest Gallo Clinic and Research Center Institutional Animal Care and Use Committee and UC Berkeley Animal Care and Use Committee. BDNF Val68Met mice were generated by the Dorit Ron lab (Warnault et al., 2016). This new line of mice differs in their targeting strategy and construct from a previously established mouse model (Chen et al., 2006). The Chen et al. (2006) line is referred to as Val66Met because the human gene is used to replace the mouse gene in this line. The line described in Warnault et al., (2016) is titled as Val68Met because target codon 68 is the appropriate valine in the mouse sequence. Further differences are outlined in the discussion.

In total, we used two mouse lines for behavioral experiments: BDNF Val68Met (Warnault et al., 2016) and BDNF Val66Met mice (Chen et al., 2006). All mice used were bred in our animal facility and were co-housed on a 12 h/12 h reverse light-dark cycle (lights on at 10 P M) in an environment enriched with bedding and toys. Adult (P60-90) males and females were used for these experiments with roughly equal sex proportions (see Supplementary Fig. 1 for total n and behavioral performance divided by sex).

**Fig. 1:**
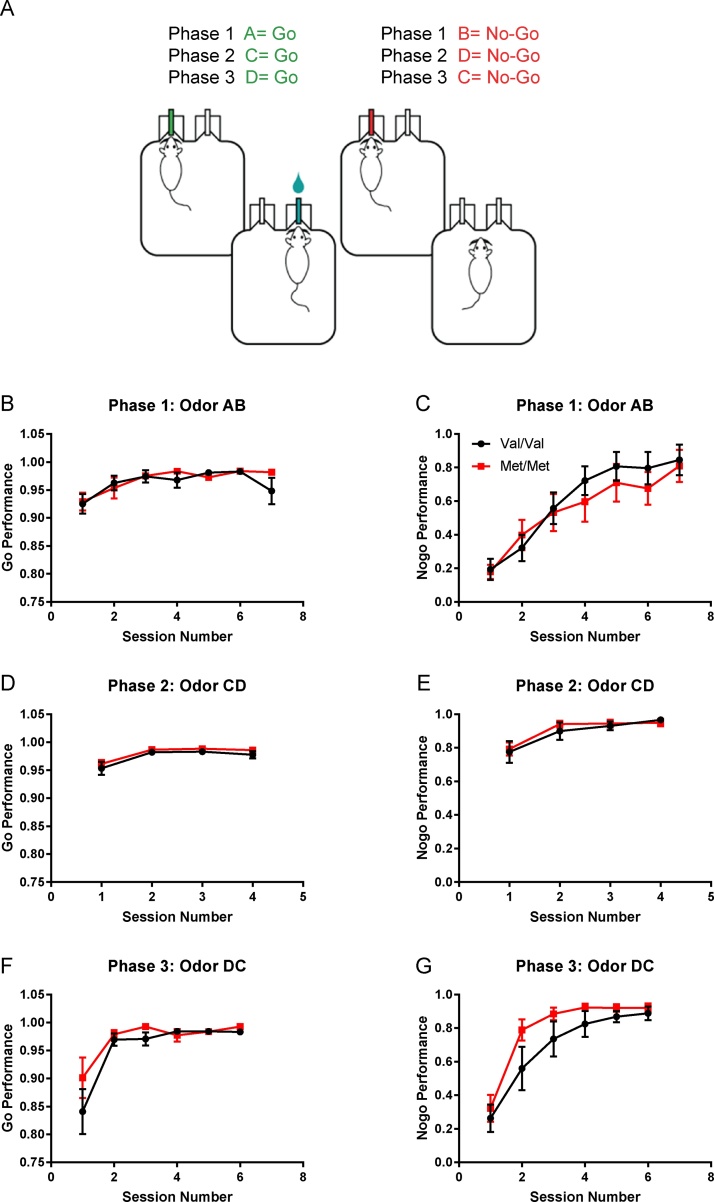
BDNF Met/Met mice learn a go/no-go task at rates comparable to Val/Val littermates, but show faster acquisition of a reversal. A, Schematic of the go/no-go task. The task had three phases: In phase 1 (shaping) mice learned the task by responding to odorants A (go cue) and B (no-go cue); In phase 2 (training) new odorants C (go cue) and D (no-go cue) were introduced; In phase 3 (reversal), odorants C and D were reversed so that C became the “no-go” cue and D became the “go” cue. B, C, Val/Val (n = 10) and Met/Met (n = 10) mice learned the task at similar rates in go and no-go performance (% correct) during the initial shaping session (A = go cue, B = no-go cue). D, E, They also performed comparably during session 2 when novel odorants C (go) and D (no-go) were introduced. F, G, In phase 3, when go and no-go odors were reversed (DC), Val/Val and Met/Met mice differed in their no-go performance (% no-go correct): A two-way ANOVA (% correct in no-go) showed a significant main effect of genotype and session number (genotype: F(1,84) = 4.03, p = 0.048, session: F(5,84) = 14.05, p < 0.0001) but no significant interaction between the two (F(5,84) = 0.35, p = 0.88). Met/Met mice achieved >80% correct in session 3 while Val/Val mice reached >80% correct in session 4.

### 2.2 Go/no-go task

For the go/no-go and go/no-go extinction tasks, we used water as a reinforcer. Mice were water restricted for two days before and throughout behavioral training, receiving 1–2 ml per day through behavioral training, as well as supplementary water in their home cage after training as needed. Mice were maintained at 90% of their ad lib weight.

The apparatus for the automated go/no-go odor discrimination task and extinction task was 5″x7″x5.″ The initiation port was located in the middle of one wall, and two choice ports were located 2.5″ to the left and right of the initiation port (center to center), but only the center port and the right port were made available during the task. An infrared photodiode/phototransistor pair was placed on either side of the port to report the times of port entry and exit (Island Motion). The water valves (Neptune Research) were calibrated to deliver a volume of water (2 μl) for rewarded choices.

Mice learned to nose poke in the center (initiation) port for odorant cue to initiate a trial. White LED lights on both the left and right ports were turned on for 3 s to indicate reward availability. Cues indicated either “go” to the right port to receive water, or “no-go” with a 30% probability of receiving a “no-go” cue. In a no-go trial, mice were required to make no response for 3 s to complete a correct trial. Incorrect trials initiated a 5 s time-out. Mice were not required to return back to a specific location between trials and could initiate the next trial immediately after collecting a reward after a go cue or inhibiting a response for 3 s after a no-go cue. The Go/No-go task included three phases: in the first phase (shaping) the animals learned the task by responding to odorants A (go cue) and B (no-go cue); in the second phase (training) new odorants C (go cue) and D (no-go cue) were introduced; in the final phase (reversal), odorants C and D reversed contingency so that C became the “no-go” cue and D the “go” cue. Odorants used were as follows: cinnamon (A), vanilla (B), bay (C), and basil (D). Animals were trained in each phase until they reach criterion of 80% correct trials.

### 2.3 Extinction task

The extinction task was similar to the go/no-go task above, but a separate cohort of mice were trained in this task. The first two phases of the task were identical to that of the go/no-go task. In the third phase, odorants C and D were still delivered after a center nose-poke, but after 400 priming trials all subsequent trials were unrewarded and no time-out was initiated. The numbers of trials completed (nose-poke in cue port followed by water port or “no-go” response) were measured. Odorants used and training criterion were the same as in phase 2 above.

### 2.4 Multiple choice discrimination and reversal task

For the multiple choice discrimination and reversal task we used cereal fragments as a reinforcer for food restricted mice. A separate cohort of adult mice were used in this task. Food restriction began two days before behavioral pre-training. During food restriction and behavioral testing, mice were maintained at 90% of their ad lib weight. Water was freely available both in the homecage and in the maze during all phases of behavioral testing.

The 4-choice maze was a square box 12″×12″×9″ with 4 internal walls measuring 3″ wide which partially divided the four quadrants. Odor stimuli were presented in ceramic pots. All pots were sham baited with a Honey Nut Cheerio (General Mills, Minneapolis, MN) secured underneath a mesh screen at the bottom. A 6″ diameter removable cylinder fit in the center of the maze and was lowered between trials (after a digging response) to isolate the mouse from the rest of the maze. This cylinder was also used as a start box.

The 4-choice odor discrimination and reversal task was adapted from Kim and Ragozzino (2005). Training took place over three days after an initial two days of food restriction. On the first day, the animals were habituated to the arena and new food. Fragments of Honey Nut Cheerio (approximately 10 mg each) were placed inside of four empty digging pots, one in each of the four quadrants. The mice were allowed to explore the maze and consume the cereal pieces for 30 min. Pots were rebaited every 10 min. On the second day of pre-training, mice were taught to find cereal fragments buried in pine wood shavings (Hartz Mountain Corporation, Secaucus, NJ). One pot with increasing amounts of wood shavings covering the cereal reward was used in this shaping phase. The quadrant containing the pot was alternated in each trial and all quadrants were rewarded equally. Trials were untimed and most animals retrieved the reward in the 12 total shaping trials within one hour. On the third day, the animals were tested. During the initial discrimination phase, the animal had to discriminate among four initial odors (anise, clove, litsea and thyme) and learn which one was associated with a buried cereal reward (anise). On the first trial of the discrimination phase (but not in the reversal phase) the animals were given a single ‘sample’ trial in which a pot with the rewarded scent was placed in the center and the animal was allowed to retrieve the cereal reward (this was the only difference from our previous protocol, Johnson and Wilbrecht, 2011). After this sample trial, the discrimination phase commenced. Each trial began with the mouse confined to the central start cylinder, which was equidistant to all the odor pots. Timing began when the cylinder was lifted. A trial was terminated if no choice was made within three minutes and was recorded as an omission. ***Criterion*** was met when the animal completed 8 out of 10 consecutive trials correctly. The stimulus presentation was pseudo-randomized such that an odor was never in the same quadrant two trials in a row.

Once criterion was met in the discrimination phase, the animal moved on to the reversal phase immediately within the same session. All shavings were replaced with new shavings to prevent discrimination via unintended cues. A previously used non-rewarded odorant (clove) became the rewarded odorant. The odor thyme (which was not rewarded) was swapped out for a novel odor (eucalyptus, also not rewarded) as a distractor. ***Perseverative errors*** were defined as trials in which the mouse dug in the pot of the previously rewarded odor (anise) before getting one correct trial (in clove). ***Regressive errors*** were trials in which the mouse dug in the pot of previously rewarded odor after the first correct trial in reversal. To complete the reversal, the mouse had to reach criterion by completing 8 out of 10 consecutive trials correctly. Mice typically completed both discrimination and reversal phases within three hours.

### 2.5 Statistics

Statistical analyses were conducted using Graphpad Prism for two-way ANOVA and Student’s t-tests. When data were found not to be normally distributed using a D'Agostino and Pearson omnibus test, we used a Mann-Whitney non-parametric test for comparison. Statistical significance was set at p < 0.05 for all analyses.

## 3 Results

### 3.1 Go/No-Go Task

Previous studies have suggested that human carriers of the Met allele make fewer errors in a go/no-go task when compared to homozygous Val individuals (Beste et al., 2010a). To determine if the same was true in Val68Met knock-in mice we tested adult mice (P60-90) on an automated odor discrimination go/no-go task (Fig. 1A). In this task, mice were trained to nose-poke in a center port for odorant cue (either a “go” or “no-go” cue) and then move to an adjacent port to receive water. Water reward was given for a correct “go” response and a 5 s time-out was given for an incorrect “no-go” response. There was a 30% probability of receiving a “no-go” cue. A correct “no-go” response required 3 s of withholding nose pokes and was not rewarded. This task had three phases: a shaping phase (Phase 1) where the animals learned the task with odorants A (“go” cue vanilla) and B (“no-go” cue cinnamon); a training phase (Phase 2) where novel odorants C (“go” cue bay) and D (“no-go” cue basil) were introduced; and a reversal phase (Phase 3) where D became the “no-go cue” and C the “go” cue.

We found that homozygous Val and Met littermates (P60-90) performed similarly in go performance (% of go trials correct) in all three phases of the task (Fig. 1B,D,F)**(**BDNF Val68Met line: Val/Val n = 10, Met/Met n = 10; Phase 1: genotype: F(1115) = 0.64, p = 0.42, session: F(6115) = 4.51, p = 0.0004, interaction: F(6115) = 0.70, p = 0.65; Phase 2: genotype: F(172) = 2.37, p = 0.13, session: F(372) = 9.99, p < 0.0001, interaction: F(372) = 0.049, p = 0.99; Phase 3: genotype: F(184) = 1.46, p = 0.23; session: F(584) = 8.06, p < 0.0001, interaction: F(584) = 0.57, p = 0.72).

In no-go performance, homozygous Val and Met littermates showed similar performance (% no-go correct each session) in the first two phases of the task, learning to avoid responding to the no-go cue with comparable accuracy (Fig. 1C,E) (Phase 1: genotype: F(1115) = 0.96, p = 0.33, session: F(6115) = 12.37, p < 0.0001, interaction: F(6115) = 0.29, p = 0.94; Phase 2: genotype: F(172) = 0.31, p = 0.58, session: F(372) = 9.18, p < 0.0001, interaction: F(372) = 0.21, p = 0.89). However, on the reversal phase of the task Met homozygous animals took fewer sessions to reach an 80% correct criterion, than Val homozygous mice did (Fig. 1G). A two-way analysis of variance (ANOVA) showed a significant main effect of genotype and session number on no-go performance (% no-go correct) (genotype: F(184) = 4.03, p = 0.048, session: F(584) = 14.05, p < 0.0001, interaction: F(584) = 0.35, p = 0.88) (Fig. 1G).

### 3.2 Extinction task

**Fig. 2:**
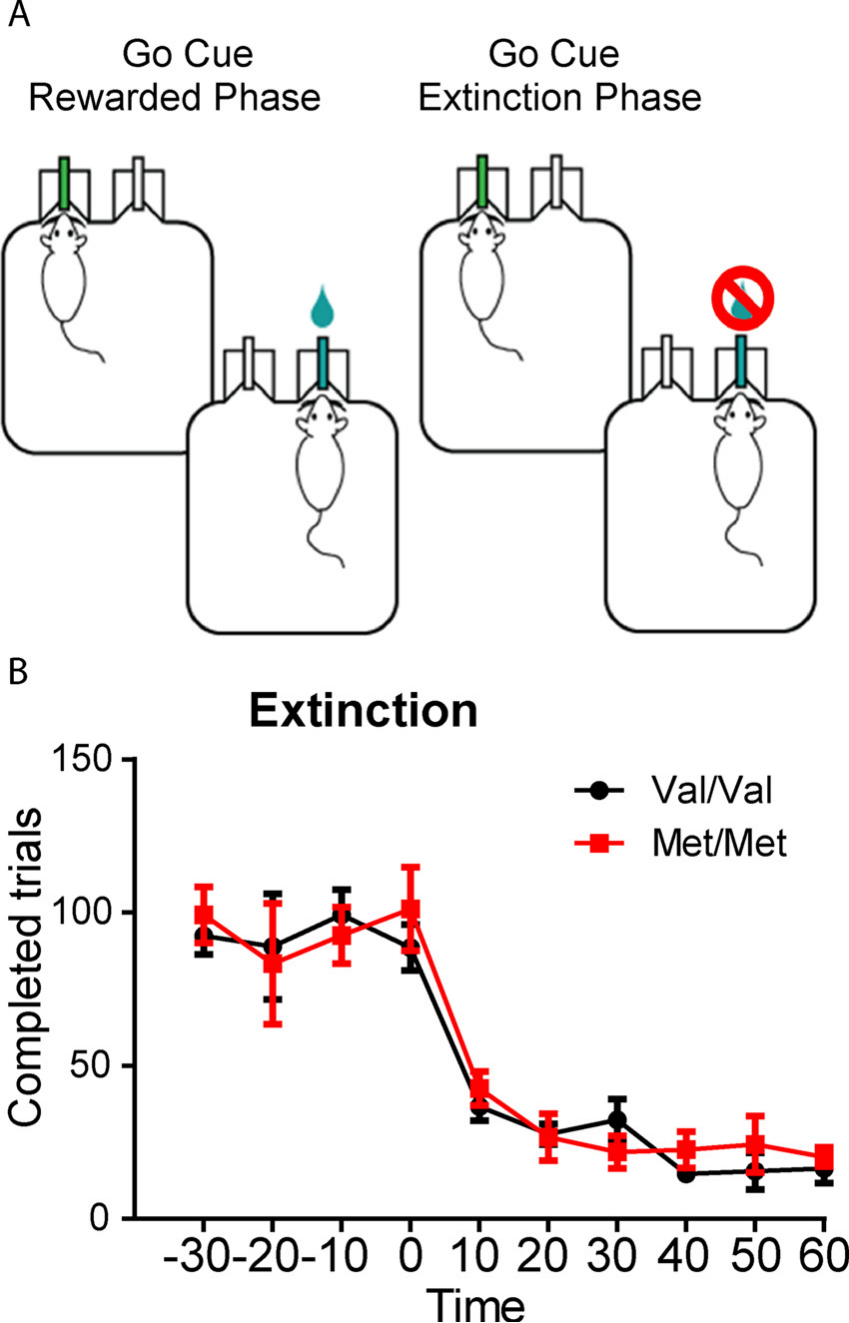
BDNF Met/Met mice extinguish a go/no-go task at comparable rates. A, Schematic of the extinction training for the ‘go’ cue, which ceased to be rewarded at time 0. A no-go cue was also continuously presented but is not shown in schematic. B, After reaching stable performance above 80% criterion, Met/Met (n = 6) and Val/Val mice (n = 8) showed comparable rates of extinction. Shown as number of completed trials. Genotype F(1,12) = 0.14, p = 0.71, time: F(9,108) = 39, p < 0.0001, interaction: F(9,108) = 0.45, p = 0.91).

In order to determine whether the more efficient reversal in Met animals was due to faster rates of extinction learning, we tested a new cohort of Val68Met littermates on an extinction task using the go/no-go paradigm (BDNF Val68Met line: Val/Val n = 8, Met/Met n = 6). The first two phases of the task were identical to the go/no-go task, above. On the final phase of the task animals were given 30 min of “maintenance” trials (where water reward was made available for correct “go” trials and a time-out for incorrect “no-go” responses) followed by one hour of within-session extinction trials (where nose-poke responses had no consequence and water delivery was unavailable) (Fig. 2A). We found similar extinction rates for both homozygous Val and Met mice examining number of completed trials (nose-poke followed by water port or “no-go” response: genotype: F(112) = 0.14, p = 0.71, time: F(9108) = 39, p < 0.0001, interaction: F(9108) = 0.45, p = 0.91) (Fig. 2B). These experiments suggest that differences in extinction learning do not explain differences in reversal learning found in BDNF Val68Met mice.

### 3.3 Multiple choice discrimination and reversal task

**Fig. 3:**
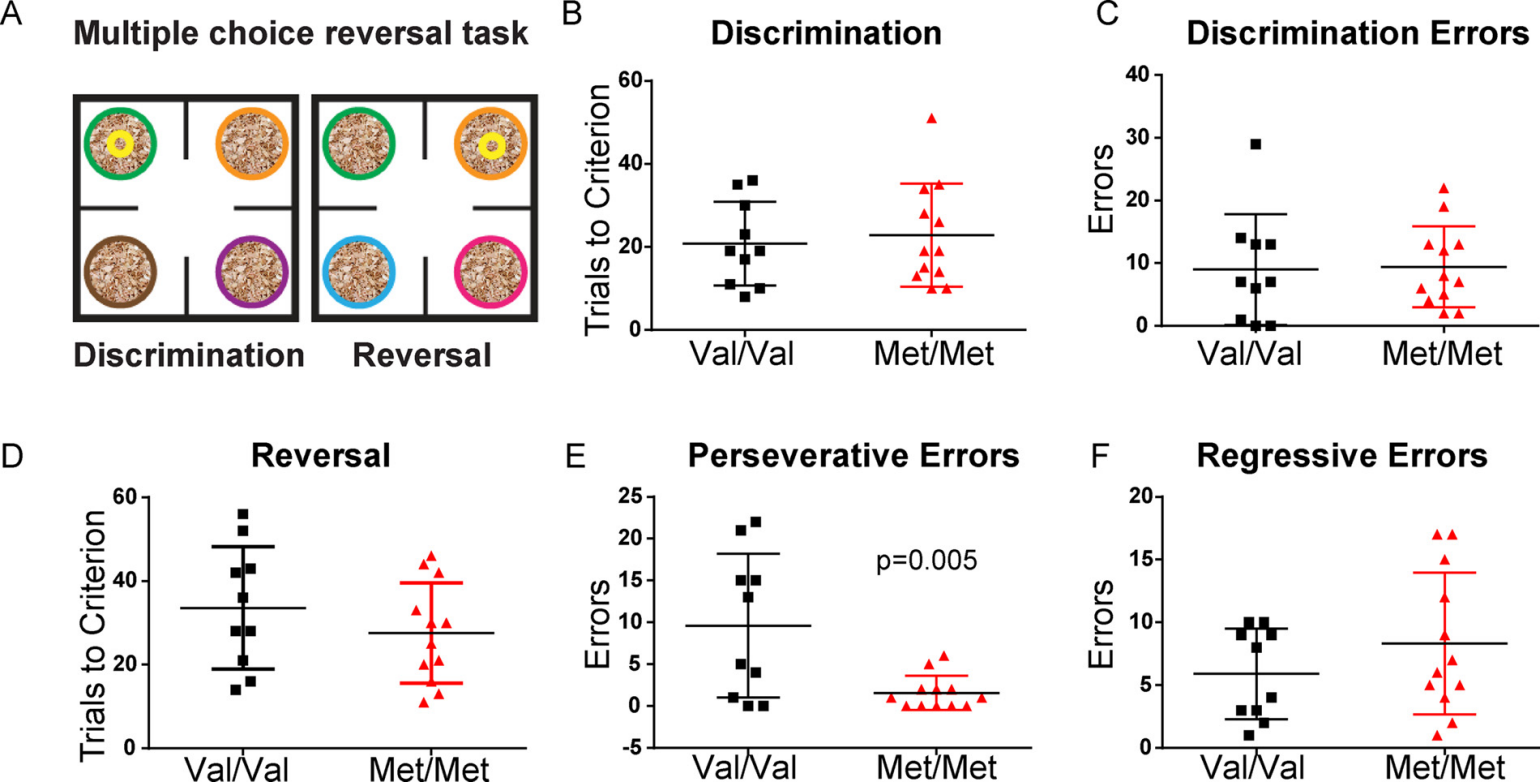
BDNF Met/Met mice learn a multiple choice discrimination task at rates comparable to Val/Val littermates, but show fewer perseverative errors in acquisition of a reversal. A, Schematic of the task. Scented shavings were introduced in 4 pots. During the initial discrimination training animals learned to discriminate odors in order to find a buried food reward. Pots were shifted after each trial and the discrimination phase ended when the mouse retrieved the reward in 8 out of 10 consecutive trials. During the reversal phase, immediately following discrimination, a previously unrewarded odor predicted the location of the reward and a novel odor was introduced. B, Met/Met (n = 12) and Val/Val (n = 10) mice took similar number of trials to reach criterion in the discrimination phase (p = 0.68), and C, made a similar number of errors (t(20) = 0.13, p = 0.90). D, In the reversal phase, trials to criterion score was comparable between groups (t(20) = 1.1, p = 0.3). E, On the way to reaching criterion, the number of perseverative errors made were fewer in Met/Met compared to Val/Val (t(20) = 3.14, p = 0.005). F, Regressive errors, made after 1 correct, were comparable between genotypes (t(20) = 1.18, p = 0.25).

To test learning and reversal under greater cognitive load and with a different modality of reinforcement, we next tested mice on a 4-choice odor discrimination task (Fig. 3A). This task has been used previously to test behavioral flexibility in rats (Ragozzino and Rozman, 2007) and mice (Johnson and Wilbrecht, 2011). During the discrimination phase of this task animals were taught to dig for buried food reward in pots with differently scented shavings. Only one scent was rewarded and pots shiftedlocation after each trial. Each phase was complete when the animal reached 8 out of 10 correct trials. We found that adult Val/Val and Met/Met mice learned the discrimination task with similar trials to criterion (Val/Val n = 10, Met/Met n = 12, t(20) = 0.42, p = 0.68) with similar total errors (t(20) = 0.13, p = 0.90) (Fig. 3B,C). However, during the reversal phase, in which a previously unrewarded odor predicted the location of the reward, Met/Met mice made significantly fewer perseverative errors back to the originally rewarded odor before their initial discovery of the new location of the reward (t(20) = 3.14, p = 0.005) (Fig. 3E). Regressive errors, defined as errors back to the originally rewarded odor after the new reward contingency was discovered once, were not different between groups (t(20) = 1.18, p = 0.25) (Fig. 3F).

In addition to comparing total trials to criterion and errors in this task, we also compared the latency for Val/Val and Met/Met mice to make a choice to dig in the task. We found that there were no significant differences in time to make a correct choice or incorrect choice between genotypes in either the discrimination phase (data not shown; correct choice latency (seconds, mean ± SEM): Val/Val = 44.68 ± 9.61, Met/Met = 36.12 ± 3.65; incorrect choice latency: Val/Val = 69.16 ± 19.34, Met/Met = 58.05 ± 9.26; Two-way ANOVA: genotype: F(140) = 0.77, p = 0.39, trial type: F(140) = 4.27, p = 0.045, interaction: F(140) = 0.013, p = 0.91) or the reversal phase (data not shown; correct choice latency (seconds, mean ± SEM): Val/Val = 30.51 ± 4.87, Met/Met = 33.79 ± 6.66; incorrect choice latency: Val/Val = 45.35 ± 7.28, Met/Met = 40.78 ± 6.73; Two-way ANOVA: genotype: F(140) = 0.0097, p = 0.92, trial type: F(140) = 2.78, p = 0.10, interaction: F(140) = 0.36, p = 0.55).

**Fig. 4:**
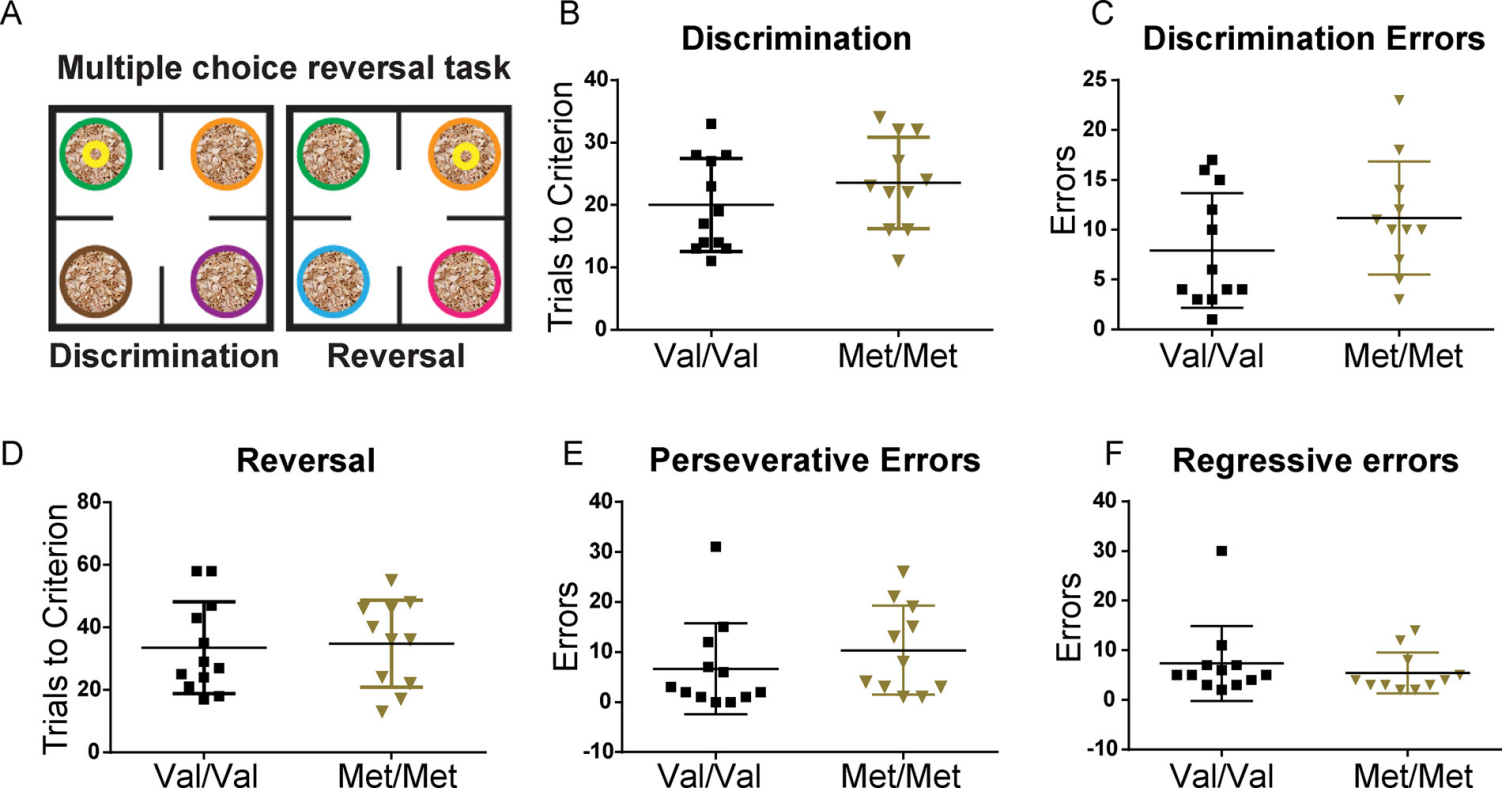
BDNF val66met mice from an alternate line (Chen et al., 2006) commonly studied as an anxiety model, do not show more flexible reversal learning. A, Schematic of the task. B, Met/Met (n = 11) and Val/Val (n = 12) mice took similar number of trials to reach criterion in the discrimination phase (t(21) = 1.14, p = 0.27), and C, made a similar number of errors (t(21) = 1.37, p = 0.19). D, In the reversal phase Trials to criterion (t(21) = 0.22, p = 0.83), perseverative errors (U = 43.50, p = 0.17) and regressive errors (U = 51.50, p = 0.38) did not differ by genotype.

In follow up, we tested an alternate line of BDNF Val66Met mice (Chen et al., 2006) in the same multiple choice discrimination and reversal task. Met homozygotes from this BDNF Val66Met line show enhanced anxiety-like behavior (Chen et al., 2006), which is not observed in the BDNF Val68Met line (Warnault et al., 2016) used to generate the data for Fig. 1, Fig. 2, Fig. 3. Using this alternate line (Chen et al., 2006), we found no significant difference in the performance between genotypes in the discrimination and reversal phase of the task in terms of trials to criterion, and perseverative and regressive errors in reversal (Fig. 4) (Discrimination phase: trials to criterion: t(21) = 1.14, p = 0.27, discrimination errors: t(21) = 1.34, p = 0.19; Reversal phase: trials to criterion: t(21) = 0.22, p = 0.83, reversal errors: t(21) = 0.48, p = 0.64, perseverative errors: U = 43.50, p = 0.17, regressive error: U = 51.50, p = 0.38). We also found no difference between the WT Val/Val groups from the two lines (Discrimination phase: trials to criterion: t(20) = 0.21, p = 0.83, discrimination errors: t(20) = 0.35, p = 0.73; Reversal phase: trials to criterion: t(20) = 0.02, p = 0.99, perseverative errors: U = 48, p = 0.44; regressive errors: U = 58, p = 0.91).

## 4 Discussion

We find that BDNF Val68Met knock-in mice raised in semi-enriched conditions perform similar to Val/Val WT littermates in tasks that tax their ability to learn. However, Met/Met mice are more flexible than Val/Val in updating their performance after a contingency reversal in a go/no-go task and in a multiple-choice odor based task.

Closer analyses of the multiple choice reversal data show Met/Met mice made fewer perseverative errors but not fewer regressive errors. Perseverative error rate could be affected by several different cognitive mechanisms: sensitivity to learning from negative feedback, differences in use of an explore versus exploit choice policy, and efficiency of behavioral inhibition or extinction. A lack of difference in regressive errors suggest both genotypes showed similar efficiency in learning from positive feedback and/or behavioral inhibition once the new correct choice was discovered. Behavioral inhibition may also be comparable between the genotypes, because both homozygous Val and Met mice made a similar number of “no-go” errors in a go/no-go task. Also, homozygous Val and Met mice showed similar appetitive extinction rates. This leaves learning from negative feedback and differences in explore versus exploit choice policy as more likely candidates for the difference in genotypes.

Our data suggest behavioral inhibition and appetitive extinction can be dissociated from flexibility in reversal learning, suggesting some mechanisms supporting these functions are independent (Izquierdo et al., 2017). Lesion, inactivation, and stimulation studies suggest different PFC subregions may support these different processes with dorsal PFC and orbital frontal cortex supporting reversal (Ragozzino and Rozman, 2007; Bissonette et al., 2008; Johnson and Wilbrecht, 2011) and medial infralimbic PFC supporting extinction learning (Sparta et al., 2014; Gourley and Taylor, 2016).

The circuit differences underlying the enhanced flexibility and plasticity of BDNF Val68Met knock-in mice are likely to be the result of reduced activity dependent release of BDNF. BDNF is thought to play a role in the maturation of inhibitory circuits that can regulate sensitive periods in the neocortex (Huang et al., 1999; Abidin et al., 2008; Werker and Hensch, 2015). BDNF from cortical terminals may also regulate striatal circuits and flexibility in drug-seeking behavior (Logrip et al., 2009; Jia et al., 2010; Warnault et al., 2016) and in the context of stress (Graybeal et al., 2011).

We speculate that higher levels of flexibility and exploratory behavior observed in juvenile mice in the same multiple choice reversal task (Johnson and Wilbrecht, 2011) may persist in the juvenile form in adult BDNF Met mice (Val68Met line) due to lower activity dependent release of BDNF. In future studies, it will be important to determine how circuit development is altered in BDNF Val68Met mice, particularly in cortical-striatal circuits known to support flexibility in reversal learning.

Our data conflict with previous reports that showed impairment in appetitive learning and in homozygous Met mice and enhanced appetitive extinction in heterozygous Val/Met mice (Briand et al., 2012). These studies used the Chen et al. (2006) BDNF Val66Met line. In follow up we found that homozygous Met mice from the Chen et al. (2006) BDNF Val66Met line mice raised in our colony under semi-enriched conditions were not impaired in multiple choice discrimination learning and did not show faster reversal performance compared to Val homozygotes. These data may represent broader differences between this more recent BDNF Val68Met line (Warnault et al., 2016) and the previously established line (Chen et al., 2006). Notably, these lines also differ in their anxiety phenotype (Warnault et al., 2016; Chen et al., 2006). We have previously discovered that early maternal separation stress, which is known to enhance anxiety-like behavior in mice also reduces cognitive flexibility in the multiple choice foraging task in juvenile mice (Thomas et al., 2016). Greater anxiety-like behavior in the Chen et al. (2006) line (in either dams or offspring) may counteract enhanced flexibility, while the Warnault et al. (2016) line shows no differences in anxiety-like behavior between genotypes (Warnault et al., 2016).

The two BDNF knock in lines intended to mimic the human BDNF val66met polymorphism also differ in their targeting strategy and construct. Chen et al. (2006) used the human BDNF coding sequence, replacing the Valine at position 66 by a Methionine by introducing a single point mutation, whereas Warnault et al. (2016) introduced two point mutations to produce the same substitution. It is important to note that Chen et al. (2006) replaced the endogenous mouse BDNF sequence with the human sequence, whereas Warnault et al. (2016) mutated the mouse BDNF sequence. Finally, Chen et al. (2006) added a carboxy-terminal Histidine repeats tag (His tag), whereas the Warnault et al. (2016) sequence did not contain additional unrelated amino acids. His tag has been shown to alter the biochemical properties and activity of recombinant proteins (Wu and Filutowicz, 1999; Panek et al., 2013). Thus, it is plausible that the His tag changed the confirmation and function of the BDNF polypeptide by for example masking a protein interaction site. Moreover, His tag increases the stability of proteins in heterologous systems (Khan et al., 2012). Thus, it is possible that the differences in the behavioral phenotypes in the two mouse lines could be due to divergent half-life of the BDNF polypeptide.

The current data show that Met homozygotes can show greater flexibility in specific contexts of reversal. Our data are interesting to compare to previous evidence that Met homozygotes from the Warnault et al. (2016) line are less flexible than Val homozygotes in the context of drug-related behavior. Met homozygotes show aversion resistant alcohol intake in a model of long-term binge drinking (Warnault et al., 2016), meaning that they are more likely to continue to binge drink alcohol even after it is laced with a bitter quinine solution. These observations showing divergent flexibility phenotypes in different contexts (after a timeout or absence of an expected reward versus quinine laced ethanol) could simply be due to independent brain changes and mechanisms. However, they are also consistent with more global gene-environment interaction models, which suggest that specific polymorphisms, instead of directly predicting risk or resilience, confer a differential responsiveness to the environment (Belsky et al., 2009; Casey et al., 2009). For example, a recent longitudinal study that looked at children that were raised with either quality foster care or reared in an institution, showed that children with the BDNF Met allele (in combination with other risk/plasticity alleles) demonstrated the highest level of indiscriminant behavior (unrestrained social boundaries) in the institutional setting and the lowest level of indiscriminant behavior in the foster care environment. Val/Val children however, demonstrated little difference in levels of indiscriminant behavior in either environment (Drury et al., 2012). This study is in line with many recent studies that suggest that ‘vulnerability’ genes such as the BDNF Met polymorphism may predict greater responsiveness to both positive and negative environments (Belsky et al., 2009; Casey et al., 2009; Drury et al., 2012; Gerritsen et al., 2012). The animals in this study were all raised in an environment enriched with bedding and toys with careful monitoring of light cycle and handling. Housing and social variables may prove significant in determining our results and may explain discrepancies in the human and animal literature. Further work in deprived or harsh environments could be used to test this model. In future work, it will also be interesting to test which domains of learning and neural circuits are sensitive to this gene x environment interaction while others, such as appetitive extinction learning, may be insensitive.

In conclusion, a relatively new mouse model of the human BDNF Val66Met polymorphism, shows no evidence of cognitive impairment in discrimination learning and enhanced flexibility in reversal learning in two different behavioral paradigms. These data are consistent with the hypothesis that BDNF val66met polymorphism is a “plasticity allele,” rather than simply a “risk allele,” and may confer positive effects in neutral or positive environments (Belsky et al., 2009).

## Conflict of interest

The authors declare no conflicts of interest.

## References

[bib0005] Abidin I., Eysel U.T., Lessmann V., Mittmann T. (2008). Impaired GABAergic inhibition in the visual cortex of brain-derived neurotrophic factor heterozygous knockout mice. J. Physiol. (Lond.).

[bib0010] Alfimova M.V., Korovaitseva G.I., Lezheiko T.V., Golimbet V.E. (2012). Effect of BDNF Val66Met polymorphism on normal variability of executive functions. Bull. Exp. Biol. Med..

[bib0015] Angelucci F., Brene S., Mathe A.A. (2005). BDNF in schizophrenia, depression and corresponding animal models. Mol. Psychiatry.

[bib0020] Bath K.G., Lee F.S. (2006). Variant BDNF (Val66Met) impact on brain structure and function. Cogn. Affect Behav. Neurosci..

[bib0025] Belsky J., Jonassaint C., Pluess M., Stanton M., Brummett B., Williams R. (2009). Vulnerability genes or plasticity genes?. Mol. Psychiatry.

[bib0030] Beste C., Baune B.T., Domschke K., Falkenstein M., Konrad C. (2010). Paradoxical association of the brain-derived-neurotrophic-factor val66met genotype with response inhibition. Neuroscience.

[bib0035] Beste C., Kolev V., Yordanova J., Domschke K., Falkenstein M., Baune B.T., Konrad C. (2010). The role of the BDNF Val66Met polymorphism for the synchronization of error-specific neural networks. J. Neurosci..

[bib0040] Biskupska J., Borowiak K.S., Karlin-Grazewicz K., Janus T., Waloszczyk P., Potocka-Banas B., Machoy-Mokrzynska A., Ossowski A., Ciechanowicz A. (2013). Estimation of BDNF gene polymorphism and predisposition to dependence development for selected psychoactive compounds: genetic aspects of addiction with the selected drugs, amphetamine, tetrahydrocannabinol and opiates. Hum. Exp. Toxicol..

[bib0045] Bissonette G.B., Martins G.J., Franz T.M., Harper E.S., Schoenbaum G., Powell E.M. (2008). Double dissociation of the effects of medial and orbital prefrontal cortical lesions on attentional and affective shifts in mice. J. Neurosci..

[bib0050] Briand L.A., Lee F.S., Blendy J.A., Pierce R.C. (2012). Enhanced extinction of cocaine seeking in brain‐derived neurotrophic factor Val66Met knock‐in mice. Eur. J. Neurosci..

[bib0055] Casey B.J., Glatt C.E., Tottenham N., Soliman F., Bath K., Amso D., Altemus M., Pattwell S., Jones R., Levita L., McEwen B., Magarinos A.M., Gunnar M., Thomas K.M., Mezey J., Clark A.G., Hempstead B.L., Lee F.S. (2009). Brain-derived neurotrophic factor as a model system for examining gene by environment interactions across development. Neuroscience.

[bib0060] Chen Z.Y., Jing D.Q., Bath K.G., Ieraci A., Khan T., Siao C.J., Herrera D.G., Toth M., Yang C., McEwen B.S., Hempstead B.L., Lee F.S. (2006). Genetic variant BDNF (Val66Met) polymorphism alters anxiety-related behavior. Science.

[bib0065] Cheng C.Y., Hong C.J., Yu YW Chen T.J., Wu H.C., Tsai S.J. (2005). Brain-derived neurotrophic factor (Val66Met) genetic polymorphism is associated with substance abuse in males. Mol. Brain Res..

[bib0070] Drury S.S., Gleason M.M., Theall K.P., Smyke A.T., Nelson C.A., Fox N.A., Zeanah C.H. (2012). Genetic sensitivity to the caregiving context: the influence of 5httlpr and BDNF val66met on indiscriminate social behavior. Physiol. Behav..

[bib0075] Duncan J.R. (2012). Current perspecitives on the neurobiology of durg addiction: a focus on genetics and factors regulating gene expression. ISRN Neurology.

[bib0080] Egan M.F., Kojima M., Callicott J.H., Goldberg T.E., Kolachana B.S., Bertolino A., Zaitsev E., Gold B., Goldman D., Dean M., Lu B., Weinberger D.R. (2003). The BDNF val66met polymorphism affects activity-dependent secretion of BDNF and human memory and hippocampal function. Cell.

[bib0085] Erickson K.I., Kim J.S., Suever B.L., Voss M.W., Francis B.M., Kramer A.F. (2008). Genetic contributions to age-related decline in executive function: a 10-year longitudinal study of COMT and BDNF polymorphisms. Front. Hum. Neurosc..

[bib0090] Frielingsdorf H., Bath K.G., Soliman F., Difede J., Casey B.J., Lee F.S. (2010). Variant brain-derived neurotrophic factor Val66Met endophenotypes: implications for posttraumatic stress disorder. Ann. N. Y. Acad. Sci..

[bib0095] Gajewski P.D., Hengstler J.G., Golka K., Falkenstein M., Beste C. (2011). The met-allele of the BDNF Val66Met polymorphism enhances task switching in elderly. Neurobiol. Aging.

[bib0100] Gajewski P.D., Hengstler J.G., Golka K., Falkenstein M., Beste C. (2012). The met-genotype of the BDNF Val66Met polymorphism is associated with reduced stroop interference in elderly. Neuropsychologia.

[bib0105] Gerritsen L., Tendolkar I., Franke B., Vasquez A.A., Kooijman S., Buitelaar J., Fernández G., Rijpkema M. (2012). BDNF Val66Met genotype modulates the effect of childhood adversity on subgenual anterior cingulate cortex volume in healthy subjects. Mol. Psychiatry.

[bib0110] Getzmann S., Gajewski P.D., Hengstler J.G., Falkenstein M., Beste C. (2013). BDNF Val66Met polymorphism and goal-directed behavior in healthy elderly - evidence from auditory distraction. Neuroimage.

[bib0115] Gourley S.L., Taylor J.R. (2016). Going and stopping: dichotomies in behavioral control by the prefrontal cortex. Nat. Neurosci..

[bib0120] Gratacos M., Gonzalez J.R., Mercader J.M., de Cid R., Urretavizcaya M., Estivill X. (2007). Brain-derived neurotrophic factor Val66Met and psychiatric disorders: meta-analysis of case-control studies confirm association to substance-related disorders, eating disorders, and schizophrenia. Biol. Psychiatry.

[bib0125] Graybeal C., Feyder M., Schulman E., Saksida L.M., Bussey T.J., Brigman J.L., Holmes A. (2011). Paradoxical reversal learning enhancement by stress or prefrontal cortical damage: rescue with BDNF. Nat. Neurosci..

[bib0130] Greenwald M.K., Steinmiller C.L., Sliwerska E., Lundahi L., Burmeister M. (2013). BDNF Val(66)met genotype is associated with drug-seeking phenotypes in heroin-dependent individuals: a pilot study. Addict. Biol..

[bib0135] Huang Z.J., Kirkwood A., Pizzorusso T., Porciatti V., Morales B., Bear M.F., Maffei L., Tonegawa S. (1999). BDNF regulates the maturation of inhibition and the critical period of plasticity in mouse visual cortex. Cell.

[bib0140] Izquierdo A., Brigman J.L., Radke A.K., Rudebeck P.H., Holmes A. (2017). The neural basis of reversal learning: an updated perspective. Neuroscience.

[bib0145] Jia Y., Gall C.M., Lynch G. (2010). Presynaptic BDNF promotes postsynaptic long-term potentiation in the dorsal striatum. J. Neurosci..

[bib0150] Jing D., Lee F.S., Ninan I. (2017). The BDNF Val66Met polymorphism enhances glutamatergic transmission but diminishes activity-dependent synaptic plasticity in the dorsolateral striatum. Neuropharmacology..

[bib0155] Joffe R.T., Gatt J.M., Kemp A.H., Grieve S., Dobson-Stone C., Kuan S.A., Schofield P.R., Gordon E., Williams L.M. (2009). Brain derived neurotrophic factor Val66Met polymorphism, the five factor model of personality and hippocampal volume: implications for depressive illness. Hum. Brain. Mapp..

[bib0160] Johnson C., Wilbrecht L. (2011). Juvenile mice show greater flexibility in multiple choice reversal learning than adults. Dev. Cogn. Neurosci..

[bib0165] Khan F., Legler P.M., Mease R.M., Duncan E.H., Bergmann-Leitner E.S., Angov E. (2012). Histidine affinity tags affect MSP1(42) structural stability and immunodominance in mice. Biotechnol. J..

[bib0170] Kim J., Ragozzino M.E. (2005). The involvement of the orbitofrontal cortex in learning under changing task contingencies. Neurobiol. Learn. Mem..

[bib0175] Logrip M.L., Janak P.H., Ron D. (2009). Escalating ethanol intake is associated with altered corticostriatal BDNF expression. J. Neurochem..

[bib0180] Mandelman S.D., Grigorenko E.L. (2012). BDNF Val66Met and cognition: all, none, or some? A meta‐analysis of the genetic association. Genes, Brain Behav..

[bib0185] Matsushita S., Arai H., Matsui T., Yuzuriha T., Urakami K., Masaki T., Higuchi S. (2005). Brain-derived neurotrophic factor gene polymorphism and alzheimer’s disease. J. Neural. Transm..

[bib0190] Miyajima F., Ollier W., Mayes A., Jackson A., Thacker N., Rabbitt P., Pendleton N., Horan M., Payton A. (2008). Brain-derived neurotrophic factor polymorphism Val66Met influences cognitive abilities in the elderly. Genes Brain Behav..

[bib0195] Panek A., Pietrow O., Filipkowski P., Synowiecki J. (2013). Effects of the polyhistidine tag on kinetics and other properties of trehalose synthase from deinococcus geothermalis. Acta Biochim. Pol..

[bib0200] Pattwell S.S., Bath K.G., Perez-Castro R., Lee F.S., Chao M.V., Ninan I. (2012). The BDNF Val66Met polymorphism impairs synaptic transmission and plasticity in the infralimbic medial prefrontal cortex. J. Neurosci..

[bib0205] Ragozzino M.E., Rozman S. (2007). The effect of rat anterior cingulate inactivation on cognitive flexibility. Behav. Neurosci..

[bib0210] Schofield P.R., Williams L.M., Paul R.H., Gatt J.M., Brown K., Luty A., Cooper N., Grieve S., Dobson-Stone C., Morris C., Kuan S.A., Gordon E. (2009). Disturbances in selective information processing associated with the BDNF Val66Met polymorphism: evidence from cognition, the P300 and fronto-hippocampal systems. Biol. Psychol..

[bib0215] Sparta D.R., Hovelsø N., Mason A.O., Kantak P.A., Ung R.L., Decot H.K., Stuber G.D. (2014). Activation of prefrontal cortical parvalbumin interneurons facilitates extinction of reward-seeking behavior. J. Neurosci..

[bib0220] Thomas A.W., Caporale N., Wu C., Wilbrecht L. (2016). Early maternal separation impacts cognitive flexibility at the age of first independence in mice. Dev. Cogn. Neurosci..

[bib0225] Tsai S.J., Hong C.J., Yu YW Chen T.J. (2004). Association study of a brain-derived neurotrophic factor (BDNF) Val66Met polymorphism and personality trait and intelligence in healthy young females. Neuropsychobiology.

[bib0230] Vandenberg A., Piekarski D.J., Caporale N., Munoz-Cuevas F.J., Wilbrecht L. (2015). Adolescent maturation of inhibitory inputs onto cingulate cortex neurons is cell-type specific and TrkB dependent. Front. Neural Circuits.

[bib0235] Ventriglia M., Chiavetto L.B., Benussi L., Binetti G., Zanetti O., Riva M.A., Gennarelli M. (2002). Association between the BDNF 196 A/G polymorphism and sporadic alzheimer’s disease. Mol. Psychiatry.

[bib0240] Wang C., Liu B., Long H., Fan L., Li J., Zhang X., Qiu C., Yu C., Jiang T. (2015). Epistatic interaction of BDNF and COMT on the frontostriatal system. Neuroscience.

[bib0245] Warnault V., Darcq E., Morisot N., Phamluong K., Wilbrecht L., Massa S.M., Longo F.M., Ron D. (2016). The BDNF valine 68 to methionine polymorphism increases compulsive alcohol drinking in mice that is reversed by tropomysin receptor kinase b activation. Biol. Psychiatry.

[bib0250] Werker J.F., Hensch T.K. (2015). Critical periods in speech perception: New directions. Annu. Rev. Psychol..

[bib0255] Wu J., Filutowicz M. (1999). Hexahistidine (His6)-tag dependent protein dimerization: a cautionary tale. Acta Biochim. Pol..

