# Supplementary material for: Mice engineered to mimic a common Val66Met polymorphism in the BDNF gene show greater sensitivity to reversal in environmental contingencies
Source: Dev Cogn Neurosci. 2018 May 30;34:34–41. doi: 10.1016/j.dcn.2018.05.009 (PMC6596311; doi:10.1016/j.dcn.2018.05.009)
Supplement: Supplementary file 1 [file mmc1.pdf]

Supplemental Figure 1

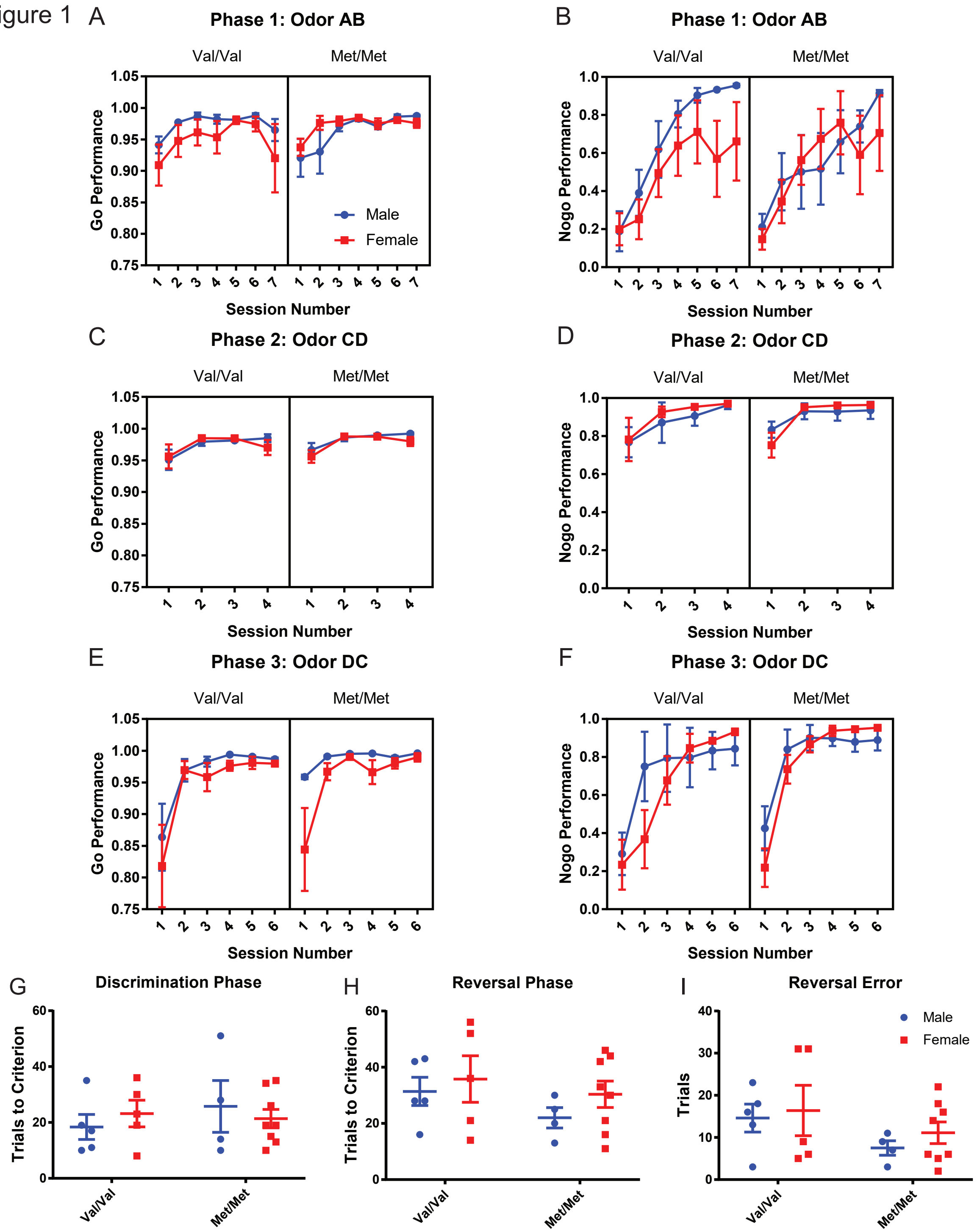

Supplemental Figure 1. We did not observe behavioral differences between sexes within Val/Val and Met/Met genotypes go/no-go task for Phase 2 and 3 and in the 4-choice discrimination and reversal. Statistics used for comparison were two-way ANOVA for sex x session. Here we report terms for sex, for brevity. No significant interactions were found.

A-F, Go/no-go task: Val/Val: Male n = 5, Female n = 5; Met/Met: Male n = 5, Female n = 5. A,B In shaping (Phase 1), male Val/Val mice showed superior go and no-go performance than female Val/Val mice (Go performance: F(1,52)=5.934, p=0.018; No-go Performance: F(1,52)=8.23,p=0.0059) but there were no sex differences in Met/Met mice for go performance (F(1,49)=1.007,p=0.32) or No-go performance (F(1,49)=0.135,p=0.71). C,E, In training (Phase 2) and reversal (Phase 3), there were no sex differences in go performance between in both Val/Val (Phase2: F(1,32)=0.0005,p=0.98; Phase3: F(1,37)=1.50,p=0.23) and Met/Met mice(Phase 2:F(1,32)=1.46,p=0.24; Phase 3: F(1,35)=3.61,p=0.066). D,F, In training (Phase 2) and reversal (Phase 3), there were no sex differences in no-go performance in either Val/Val or Met/Met genotypes. (Val/Val: Phase 2: F(1,32)=0.46,p=0.50; Phase 3: F(1,37)=0.47,p=0.50; Met/Met: Phase 2: F(1,32)=0.00015,p=0.99; Phase 3: F(1,35)=0.31,p=0.58). G-I, We did not observe sex differences in the 4-choice discrimination and reversal task in either genotype. Each point is a mouse. Val/Val: Male n = 5, Female n = 5; Met/Met: Male n = 4, Female n = 8. (Trials to criterion in Discrimination: F(1,18)=0.0017, p=0.97; Trials to criterion in Reversal: F(1,18)=1.16,p=0.30; Reversal Error: F(1,18)=0.51,p=0.49).
